# Liupao tea aqueous extract alleviates dextran sulfate sodium-induced ulcerative colitis in rats by modulating the gut microbiota

**DOI:** 10.1515/biol-2025-1106

**Published:** 2025-05-20

**Authors:** Shengjin Ming, Jinxi Ou, Ying Huang, Zhongqing Tang, Yuechao Qin, Hongxi Ma, Siling Gan, Zhongxia Li

**Affiliations:** Department of Clinical Laboratory, Wuzhou Gongren Hospital (The Seventh Affiliated Hospital of Guangxi Medical University), 1 Gaodi Road, Wuzhou, 543001, China; Department of Gastroenterology, Wuzhou Gongren Hospital (The Seventh Affiliated Hospital of Guangxi Medical University), 1 Gaodi Road, Wuzhou, 543001, China; Department of Pathology, Wuzhou Gongren Hospital (The Seventh Affiliated Hospital of Guangxi Medical University), 1 Gaodi Road, Wuzhou, 543001, China; Department of Pediatrics, Wuzhou Gongren Hospital (The Seventh Affiliated Hospital of Guangxi Medical University), 1 Gaodi Road, Wuzhou, Guangxi, 543001, China

**Keywords:** Liupao tea aqueous extract, gut microbiota, colitis, short-chain fatty acids, anti-inflammatory effect

## Abstract

Liupao tea is known for its anti-inflammatory antioxidant and regulation of gut microecological balance properties. This study aims to investigate the therapeutic effects of Liupao tea aqueous extract (LPTAE) on ulcerative colitis (UC) induced by dextran sulfate sodium (DSS) in rats. The rats were randomly divided into five groups: the Normal group, the DSS group, the LPTL group, the LPTM group, and the LPTH group. Throughout the experiment, the rats’ activity levels, stool consistency, and body weights were observed and recorded daily. After the experiment, colon length was measured, and colon tissues were collected for pathological analysis. Additionally, the colon contents were analyzed for gut microbiota composition and short-chain fatty acid (SCFA) level, while serum samples were collected to determine inflammatory and oxidative factors. The results indicated that treatment with low, medium, and high doses of LPTAE significantly inhibited weight loss, alleviated rectal bleeding, and reduced colon shortening compared to the DSS group. It also decreased the disease activity index and histopathological activity index scores in the rats. Furthermore, LPTAE reduced the levels of inflammatory cytokines such as IL-1*β*, IL-6, TNF-*α*, and malondialdehyde, while simultaneously increasing the levels of superoxide dismutase and SCFAs, including acetic acid, propionic acid, and butyric acid. 16S rDNA gene sequencing of the gut microbiota revealed that all doses of LPTAE reversed the decrease in both *α* and *β* diversities caused by UC, increased the relative abundance of beneficial bacteria such as *Lactobacillus*, Muribaculaceae, *Alloprevotella*, and *Blautia*, and decreased the levels of harmful bacteria such as *Prevotella*, *Romboutsia*, and *Bacteroides*. In summary, within the tested doses (100, 150, 250 mg/kg), LPTAE alleviated DSS-induced colitis by modulating the gut microbiota and correcting the metabolic imbalance of SCFAs.

## 1 Introduction

Ulcerative colitis (UC) is an inflammatory bowel disease (IBD) primarily characterized by inflammation of the intestinal mucosa, the main areas affected are the rectum and colon, patients typically experience symptoms such as abdominal pain, diarrhea, bloody stools, and weight loss [1]. Chronic and recurrent inflammatory flare-ups can increase the risk of UC progressing to colon cancer [2]. The exact cause of UC remains unclear, but current research suggests that its onset is closely related to factors including genetics, environmental influences, disruptions in gut microbiota, and autoimmune system disorders [3]. The prevalence of UC has been rising globally in recent years, especially in developing countries, which has made it a worldwide public health concern, it is estimated that by 2050, there could be 1.5 million IBD patients in China alone, highlighting the severity of this issue [4]. The primary treatments for UC include 5-aminosalicylic acid, sulfasalazine, mesalazine, biologics, and immunomodulators [3]. However, these medications often come with side effects and can be costly, creating a financial and physical burden for patients who require long-term treatment. Therefore, finding an economical, effective, and low-side-effect treatment for UC that encourages high patient adherence is of significant clinical importance.

Liupao tea, a well-known beverage from Guangxi, is cherished by the local population and is named after its origin in Liupao Town, Wuzhou City, Guangxi. Historical records trace the origins of Liupao tea back to a medical book from China’s Northern and Southern Dynasties, as a type of black tea, Liupao tea is celebrated for its various health benefits, including improving stomach health and digestion, alleviating heatstroke and dampness, and enhancing intestinal function. During China’s late Qing Dynasty, Liupao tea gained both domestic and international fame for its ability to alleviate gastrointestinal issues among Chinese laborers seeking work in Southeast Asia due to climate changes, this led to the tea being referred to as “life-saving tea” [5]. Liupao tea is included in the Chinese Pharmacopoeia as a medicinal tea and is rich in active compounds such as tea polyphenols, tea polysaccharides, theanine, and alkaloids, which provide anti-inflammatory and antioxidant properties [6]. Research has shown that Liupao tea can regulate oral microbiota, enhance oral tissue regeneration, and reduce mild oral inflammation [7]. It also can regulate gut microbiota, relieve airway inflammation in allergic asthma, improve hyperglycemic conditions in diabetes, and reduce fat accumulation in non-alcoholic fatty liver disease [8,9,10]. Additionally, a study conducted by Gong et al. [11] found that extracts of Liupao tea significantly promote the transport and colonization of beneficial bacteria like *Bifidobacterium* and *Lactobacillus* in the gastrointestinal tracts of normal mice, while inhibiting the growth of harmful bacteria such as *Escherichia coli* and *Enterococcus*. Liupao tea also demonstrates a remarkable ability to reverse ileal pathological damage and restore gut microbiota balance disrupted by antibiotics. Current research indicates UC is closely related to imbalances in intestinal microecology, characterized by a decrease in the abundance of probiotics and commensal bacteria and an increase in harmful bacteria [12]. One study revealed that germ-free mice developed symptoms similar to colitis after receiving fecal microbiota from UC patients, establishing a causal relationship between gut microbiota and UC [13]. Therefore, we hypothesize that Liupao tea can effectively influence UC by inhibiting inflammatory and oxidative stress responses, which may be partly linked to its effects on gut microbiota.

We evaluated the therapeutic potential of Liupao tea aqueous extract (LPTAE) in rats treated with dextran sulfate sodium (DSS), a commonly used animal model for UC [14]. DSS is a highly hydrophilic compound, and its molecular weight ranges from 36,000 to 50,000 Da, which gives it a specific binding affinity and toxicity toward colonic epithelial cells. This compound disrupts the integrity and permeability of the colonic epithelial structure, alters the characteristics of the gut microbiota, and induces UC. The DSS-induced UC model closely resembles human UC [15].

## 2 Materials and methods

### 2.1 Materials and reagents

Liupao tea was purchased from Wuzhou Liupao Tea Co., Ltd. (Qin-Yi-She-Qian tea, produced in 2019) (China). DSS (MW 40,000 Da) was acquired from Bide Pharma Technology Co., Ltd. (Shanghai, China). ELISA kits for rat IL-1*β* (batch number: BY-ER330206), IL-6 (batch number: BY-ER330219), TNF-*α* (batch number: BY-ER331063), malondialdehyde (MDA) (batch number: BY-JZF0135), and superoxide dismutase (SOD) (batch number: BY-ER334817) were obtained from Nanjing Boyan Biotech Co., Ltd. (China). The fecal genomic DNA extraction kit was sourced from Omega Bio-Tek (USA). The 2× Taq Plus Master Mix was purchased from Vazyme (China). Agencourt AMPure XP was obtained from Beckman Coulter (USA). The NEB Next Ultra II DNA Library Prep Kit was purchased from New England Biolabs (USA). The Agilent DNA 1000 Kit was sourced from Agilent (USA). The MiSeq Reagent Kit v3 (600 cycles) (PE300) was acquired from Illumina (USA). A Nanodrop 2000 Spectrophotometer was purchased from Thermo Fisher Scientific (USA). The ABI 9700 PCR Amplifier was obtained from Applied Biosystems (USA). A gas chromatograph (model: GC-2030) was acquired from Shimadzu (Japan). The mass spectrometer (model: QP2020 NX) was also purchased from Shimadzu (Japan). Standard samples of acetic acid, propionic acid, butyric acid, isobutyric acid, valeric acid, and isovaleric acid were sourced from Dr. Ehrenstorfer (Germany).

### 2.2 Experimental animals

A total of 25 healthy male Sprague-Dawley (SD) rats, each weighing between 190 and 210 g, were obtained from the Experimental Animal Center of Southern Medical University (experimental animal license number: SCXK(Yue)2021-0041). The rats were housed in the SPF-grade animal facility at the Wuzhou Institute for Food and Drug Control, where the environment was maintained at a controlled temperature of 25°C and humidity of 60%. The animals had free access to food and water throughout the study.


**Ethical approval:** The research related to animal use has been complied with all the relevant national regulations and institutional policies for the care and use of animals and has been approved by the Ethics Committee of Wuzhou Gongren Hospital (permit number: EC-2023-KY-027) as well as the Animal Ethics Committee of the Wuzhou Institute for Food and Drug Control (approval number: 20231101). We followed the guidelines established by the Animal Ethics Committee during all animal experiments.

### 2.3 Preparation of LPTAE

We employed a water extraction method to obtain an extract that closely resembles the components consumed in our daily tea. We began by weighing 100 g of Liupao tea samples and adding 1 L of ultrapure water, adhering to a tea-to-water ratio of 1:10 (g/mL). The mixture was soaked for 20 min and then boiled for 1 h. After boiling, the tea–water mixture was filtered using a 300-mesh filter cloth to produce tea decoction A. Next, we mixed the remaining tea residue with an additional 1 L of ultrapure water, boiled it for 1 h, and filtered it again using the same 300-mesh filter cloth, resulting in tea decoction B. We then combined tea decoctions A and B and poured the mixture into a stainless steel tank. The combined mixture was heated and concentrated to yield a concentrated liquid. Finally, we used a spray dryer (GEA Niro experimental spray dryer) for continuous spray drying, with a spray rate of 1 L/h, to produce a dried powder from the LPTAE. The final yield of tea powder was 16%, and it was stored at −20°C for future use.

### 2.4 Animal experiments

After a week-long acclimatization period in an SPF-grade animal facility, rats were randomly assigned to one of the five groups, each containing five individuals: the Normal group (which served as a healthy control), the DSS group (which established a UC model), the LPTL group (which received a low dose of LPTAE), the LPTM group (which received a medium dose of LPTAE), and the LPTH group (which received a high dose of LPTAE). During the first 7 days of the study, the Normal group had unlimited access to purified water, while the DSS, LPTL, LPTM, and LPTH groups were given a 5% DSS solution *ad libitum* to induce an acute UC model. In the following 7 days, the LPTL, LPTM, and LPTH groups received LPTAE solutions at dosages of 100, 150, and 250 mg/kg, respectively, with each rat receiving a 1 mL gavage. The Normal and DSS groups were given an equivalent volume of saline solution. Throughout the study, daily observations were made regarding food and water consumption, fur appearance, activity levels, and stool consistency across all groups. On day 15, the rats were humanely euthanized. Under sterile conditions, blood was collected from the abdominal veins for analysis of inflammatory and oxidative stress markers. The colons were then extracted, their lengths were measured, and approximately 5 g of colonic content was rapidly stored at −80°C for 16S rDNA gene sequencing to assess gut microbiota composition. Finally, the distal 2 cm of the colon was rinsed with 0.9% saline and fixed in 10% neutral formalin for subsequent histopathological examination of the tissue.

### 2.5 Assessment of rat body weight and disease activity index (DAI)

During the experimental period, each rat was weighed and recorded at three specific time points: on the 1st, 8th, and 15th days. Additionally, fecal consistency and the presence of fecal blood were carefully observed and documented for each subject. The DAI was calculated using a composite metric derived from the assessment of body weight loss, rectal bleeding, and fecal consistency, following the established criteria outlined in the referenced literature [16]. The body weight loss rate was determined using the formula: (initial body weight – current body weight)/initial body weight, while the relative body weight was calculated as current body weight/initial body weight. The DAI score was obtained by averaging the scores for body weight loss, fecal consistency, and rectal bleeding, calculated as follows: (body weight loss score + fecal consistency score + rectal bleeding score)/3. Specific scoring criteria are found in Table 1.

**Table 1:** DAI scoring criteria

Weigh loss	Rectal bleeding	Stool consistency	Score
0	Normal	Normal	0
0.1–5%	Small amount of blood	Loose stool	1
5–10%	Blood in stool regularly seen	Mild diarrhea	2
>10%	Blood in all stool	Diarrhea	3

### 2.6 Histopathological analysis of colon tissue

Colonic tissues were processed by being fixed in 10% neutral formalin for 3 days and then embedded in paraffin. The tissues were sectioned into thin slices with a uniform thickness of 4–5 cm. These sections were placed on a slide warmer for 10 min to promote adhesion, after which they underwent dewaxing in xylene for an additional 10 min. Following this, the sections were rinsed with graded alcohol concentrations and distilled water to remove any residual wax. Staining was performed using hematoxylin for 5 min, followed by a brief 10 s exposure to eosin. Any excess eosin was then washed away with distilled water and alcohol before the slides were air-dried and mounted with neutral gum. A microscopic examination of the colonic tissue architecture was conducted, and a histopathological score was assigned based on the severity of epithelial damage and the extent of inflammatory cell infiltration. The histopathological activity index (HAI) for the rat colon tissue was calculated as the sum of these two scores. The criteria for HAI evaluation were based on the methodology outlined in the referenced literature [17]. The specific scoring criteria are detailed in Table 2.

**Table 2:** HAI scoring criteria

Colonic epithelial damage	Inflammatory cell infiltration	Score
Normal	Normal	0
Mild goblet cell disruption	Infiltration around the crypt bases	1
Significant goblet cell disruption	Infiltration into the muscularis mucosa	2
Mild glandular disruption	Diffuse infiltration of muscularis mucosa with edema and hypertrophy	3
Significant glandular disruption	Infiltration into the submucosa	4

### 2.7 Determination of inflammatory cytokines and oxidative stress factors in serum samples

After the euthanasia of the rats, 3 mL of abdominal venous blood was collected and allowed to sit for 4 h. The blood was then centrifuged at 4,000 rpm for 10 min. The resulting serum was stored in an ultra-low temperature freezer (−80°C). The levels of IL-1*β*, IL-6, TNF-*α*, MDA, and SOD in the serum were detected using ELISA kits strictly according to the instructions provided with the kits.

### 2.8 16S rDNA gene sequencing of gut microbiota

Using the Omega Stool DNA Kit, we extracted bacterial genomic DNA from each fecal sample following the manufacturer’s protocol. The concentration and purity of the extracted DNA were evaluated with a Nanodrop 2000 spectrophotometer to ensure sample quality for downstream applications. For the amplification of bacterial 16S rDNA, we targeted the V3–V4 hypervariable regions using universal primers 338F (5′-ACTCCTACGGGAGGCAGCAG-3′) and 806R (5′-GGACTACNNGGGTATCTAAT-3′). To enable sample multiplexing, we added an 8-base pair (bp) barcode sequence to the 5′ ends of both forward and reverse primers for the distinct identification of individual samples. The synthesis of these barcoded universal primers facilitated the simultaneous processing of multiple samples. Amplification was performed on an ABI 9700 PCR system. The PCR mixture had a total volume of 25 μL and consisted of 12.5 μL of 2× Taq Plus Master Mix, 3 μL of BSA (at a concentration of 2 ng/μL), 1 μL of the forward primer (at 5 μM), 1 μL of the reverse primer (at 5 μM), 2 μL of DNA template (with a total DNA input of 30 ng), and 5.5 μL of nuclease-free water to bring the volume to 25 μL. The thermal cycling conditions were as follows: an initial denaturation step at 95°C for 5 min; 28 cycles of denaturation at 95°C for 45 s, annealing at 55°C for 50 s, and extension at 72°C for 45 s, concluding with a final extension at 72°C for 10 min. The PCR amplicons were analyzed for the correct band size using 1% agarose gel electrophoresis and subsequently purified with the Agencourt AMPure XP nucleic acid purification system. The purified PCR products were then subjected to high-throughput sequencing on the Illumina MiSeq PE300 platform, utilizing paired-end sequencing methodology. The resulting raw sequencing data were deposited in the NCBI Sequence Read Archive database, with sequencing services provided by Beijing Allwegene Technology Co., Ltd.

### 2.9 Sequencing data analysis

After obtaining sequencing data from the sequencing machine, we demultiplexed the samples based on barcode sequences using QIIME1 software (version 1.8.0). The data were then filtered and assembled using Pear software (version 0.9.6). We removed sequences with low scores below 20, as well as those containing ambiguous bases or primer mismatches. During the assembly process, we set the minimum overlap to 10 bp and the *p*-value to 0.0001. Following assembly, we utilized Vsearch software (version 2.7.1) to eliminate sequences shorter than 230 bp and to identify and remove chimeric sequences using the uchime method based on the Gold Database. The uparse algorithm in Vsearch was employed for Operational Taxonomic Units (OTU) clustering on high-quality sequences, applying a sequence similarity threshold of 97%. Next, we used the RDP Classifier algorithm to align these sequences with the Silva 128 database, setting a confidence threshold of 70% to obtain species classification information for each OTU. We then performed *α*-diversity index analysis with QIIME1 (version 1.8.0). Based on the species annotation and relative abundance results, we generated bar plots for species composition analysis using R software (version 3.6.0). For *β*-diversity analysis, we calculated the distance matrix with QIIME1 (version 1.8.0) and employed the ade4, ggplot2, and ggrepel packages in R (version 3.6.0) for principal component analysis (PCA) and non-metric multidimensional scaling (NMDS) analysis and visualization. Additionally, we conducted inter-group difference analysis using Mothur software (version 1.34.4) and performed LEfSe analysis with Python software (version 2.7).

### 2.10 Determination of short-chain fatty acids (SCFAs) in colonic contents

To prepare the fecal sample for the analysis of SCFAs, we accurately weighed 20 mg of the sample and transferred it into a 2 mL Eppendorf tube. We then added 1 mL of deionized water to the tube and vortex-mixed it for 10 s to ensure homogeneity. Next, we subjected the sample to mechanical disruption using a grinder set at 40 Hz for 4 min, followed by ultrasonication for 5 min. This ultrasonication step was repeated two more times, resulting in a total of three ultrasonication cycles. Subsequently, we centrifuged the sample at 4°C at 5,000 rpm for 20 min to pellet the solid debris. We carefully pipetted 0.8 mL of the supernatant into a fresh 2 mL Eppendorf tube. To this supernatant, we added 0.1 mL of a 50% sulfuric acid solution and 0.8 mL of an extraction solution containing 2-methylpentanoic acid as an internal standard at a concentration of 25 mg/L in methyl *tert*-butyl ether. We vortex-mixed the contents for 10 s, manually shook the mixture for 10 min, and then subjected the sample to ultrasonication in an ice-water bath for an additional 10 min to facilitate extraction. After the extraction, we centrifuged the sample again at 4°C at 10,000 rpm for 15 min to separate the phases. We allowed the sample to stand at −20°C for 30 min to promote the crystallization of any residual water. Finally, we pipetted 200 μL of the clear supernatant into a sample vial for analysis.

### 2.11 Statistical analysis

The data were analyzed and visualized using GraphPad Prism 8.0 software. Measurement data were presented as “mean ± standard deviation.” Comparisons between two groups were performed using the Student’s *t*-test, while comparisons among multiple groups were conducted using one-way analysis of variance. Spearman correlation analysis and heatmap plotting were executed using R software (version 3.6.0). *p*-values were calculated, with a *p* < 0.05 considered statistically significant.

## 3 Results

### 3.1 Effect of LPTAE intervention on symptoms of DSS-induced UC in rats

#### 3.1.1 Effect of LPTAE on the general activity status of rats

**Figure 1:**
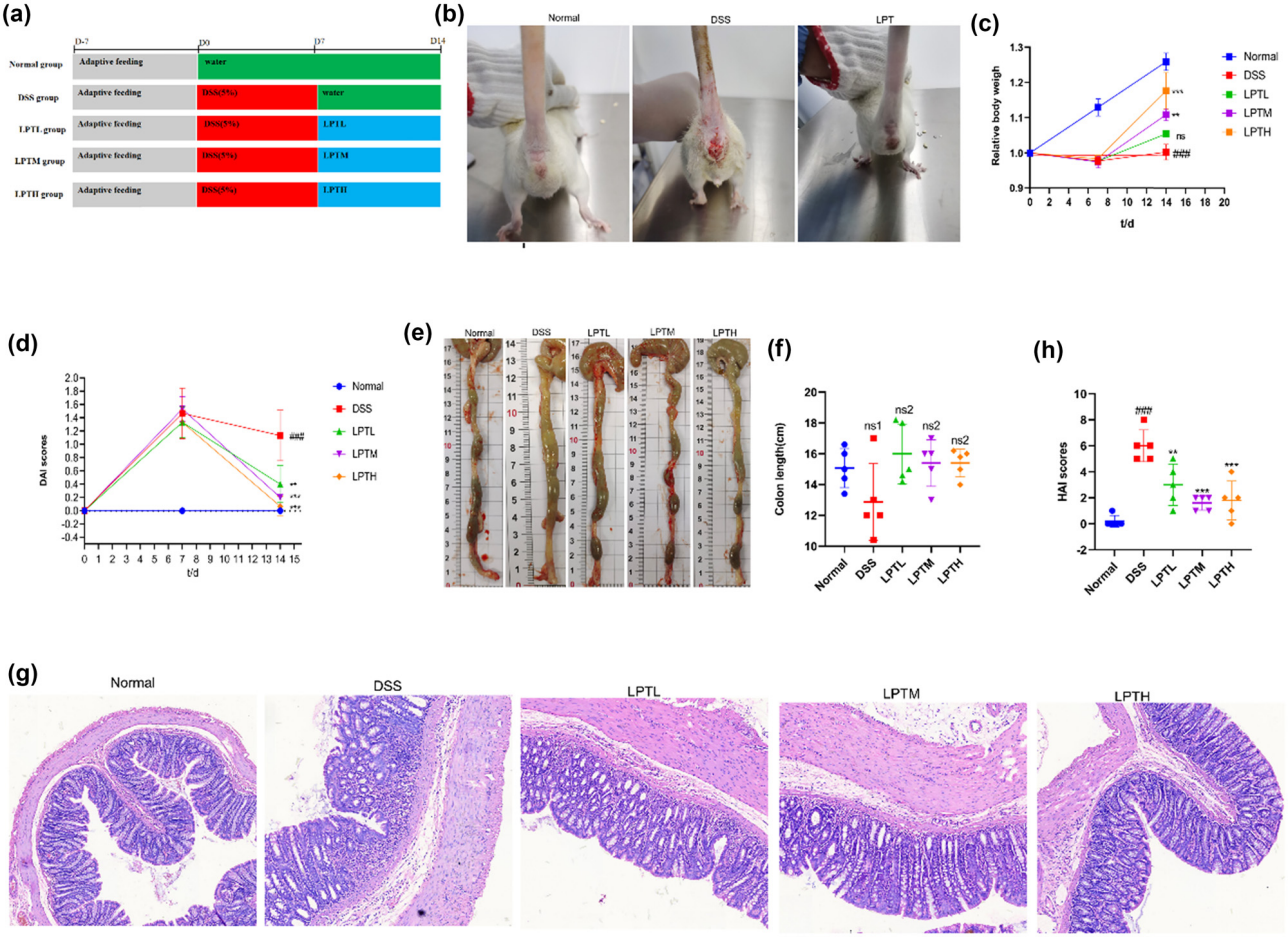
The ameliorative effect of LPATE on DSS-induced UC in rats. (a) Schematic illustration of the experimental workflow. (b) Representative photographic images of rats. (c) Percentage change in body weight over time. (d) DAI scoring. (e) Representative gross images of the colon. (f) Quantitative analysis of colon length. (g) Representative histological micrographs of colon tissues stained with hematoxylin and eosin (magnification, ×200). (h) HAI scoring of colonic morphology in rats. All data are presented as mean ± standard deviation (*n* = 5). ###*p* < 0.001 vs Normal group; **p < 0.01, ***p < 0.001 vs DSS group; ns1 denotes no statistically significant difference compared with the Normal group; ns2 denotes no statistically significant difference compared with the DSS group.

In this study, we established an animal model of acute experimental UC in rats using 5% DSS and evaluated the therapeutic efficacy of LPTAE. The experiment followed the timeline outlined in Figure 1a. After the experiment began, rats in the Normal group displayed normal behavior regarding diet, water intake, fecal consistency, coat appearance, and activity levels. In contrast, rats in the DSS, LPTL, LPTM, and LPTH groups showed reduced food intake and loose, moist feces by the second day after having free access to the 5% DSS solution. After 7 days of modeling, all rats in the model groups exhibited similar symptoms, including a loose and disheveled coat that appeared rough and dull, lethargy, clustering, somnolence, tarry feces with blood, and some rats even displayed signs of irritability, such as arched backs and bristled fur. Additionally, there was noticeable bloodstaining around the anus. Following this, we administered a 7-day intervention with LPTAE. We observed that compared to the DSS group, rats in the LPTL, LPTM, and LPTH groups showed improvements in coat glossiness, increased food and water intake, near-normalization of fecal consistency, disappearance of bloody stool symptoms, and overall behavior that approached that of the Normal group (Figure 1b).

#### 3.1.2 Effect of LPTAE on body weight and DAI scores of rats

After random grouping, no statistically significant differences in body weight were found among the groups (*p* > 0.05). Throughout the experiment, the body weight of rats in the Normal group consistently increased. However, after 7 days of unrestricted access to 5% DSS, the model groups experienced weight loss and a notable reduction in weight gain. After 7 days of LPTAE intervention, the relative body weights of rats in the LPTL, LPTM, and LPTH groups showed a dose-dependent increase compared to the DSS group, whose relative body weight remained unchanged (Figure 1c). To assess disease severity in the rats, the DAI score was utilized, which considers three indicators: percentage of weight loss, fecal consistency, and rectal bleeding. The results indicated that after 7 days of free access to 5% DSS, the DAI scores for all model groups increased significantly. Following 7 days of LPTAE intervention, the DAI scores for the LPTL, LPTM, and LPTH groups demonstrated a dose-dependent decrease compared to the DSS group (*p* < 0.01) (Figure 1d).

#### 3.1.3 Effect of LPTAE on colon length in rats

During the experiment, we observed that the colons of rats in the DSS group were swollen and thinned. Compared to the Normal group, the colon length in the DSS group was shorter, but this difference was not statistically significant (*p* > 0.05). In contrast, the colon lengths in the LPTL, LPTM, and LPTH groups showed a reversal of the shortening trend observed in the DSS group; however, the differences among these groups were also not significant (*p* > 0.05) (Figure 1e and f). We speculated that the short duration of the disease process may have contributed to these insignificant differences.

#### 3.1.4 Effect of LPTAE on histopathological findings in the colons of rats

Results from HE staining (Figure 1g) revealed that the colonic epithelial structure in the Normal group was intact, featuring abundant and well-arranged goblet cells and glands distributed uniformly, with no signs of inflammatory cell infiltration. In contrast, the DSS group showed significant destruction of the epithelial structure, marked damage and reduction of goblet cells and glands, disordered arrangement, and extensive inflammatory cell infiltration in both the mucosal and submucosal layers, indicating substantial damage to the colonic tissue. When comparing the DSS group to the groups treated with LPTAE (LPTL, LPTM, and LPTH groups), the colonic epithelial structures in these treatment groups appeared relatively intact. They exhibited less destruction and a reduction in goblet cells and glands, as well as a more regular arrangement. Additionally, the degree of inflammatory cell infiltration was significantly reduced in the LPTAE groups. The HAI scores (Figure 1h) indicated that, compared to the Normal group, the DSS group had a significantly increased HAI score (*p* < 0.001). However, following treatment with LPTAE, the HAI scores in the LPTL, LPTM, and LPTH groups were significantly lower than those in the DSS group (*p* < 0.01, *p* < 0.001).

In summary, our experimental results demonstrate that we successfully created acute experimental UC models in rats after they had free access to 5% DSS for 7 days. The key indicators of this condition included weight loss, loose stools, bloody stools, elevated DAI scores, swelling and shortening of the colon, inflammatory changes in colonic tissue, and increased HAI scores. Treatment with low, medium, and high doses of LPTAE all contributed to alleviating the symptoms of DSS-induced acute experimental UC.

### 3.2 Effect of LPTAE intervention on inflammatory response and oxidative stress system in DSS-induced rats

**Figure 2:**
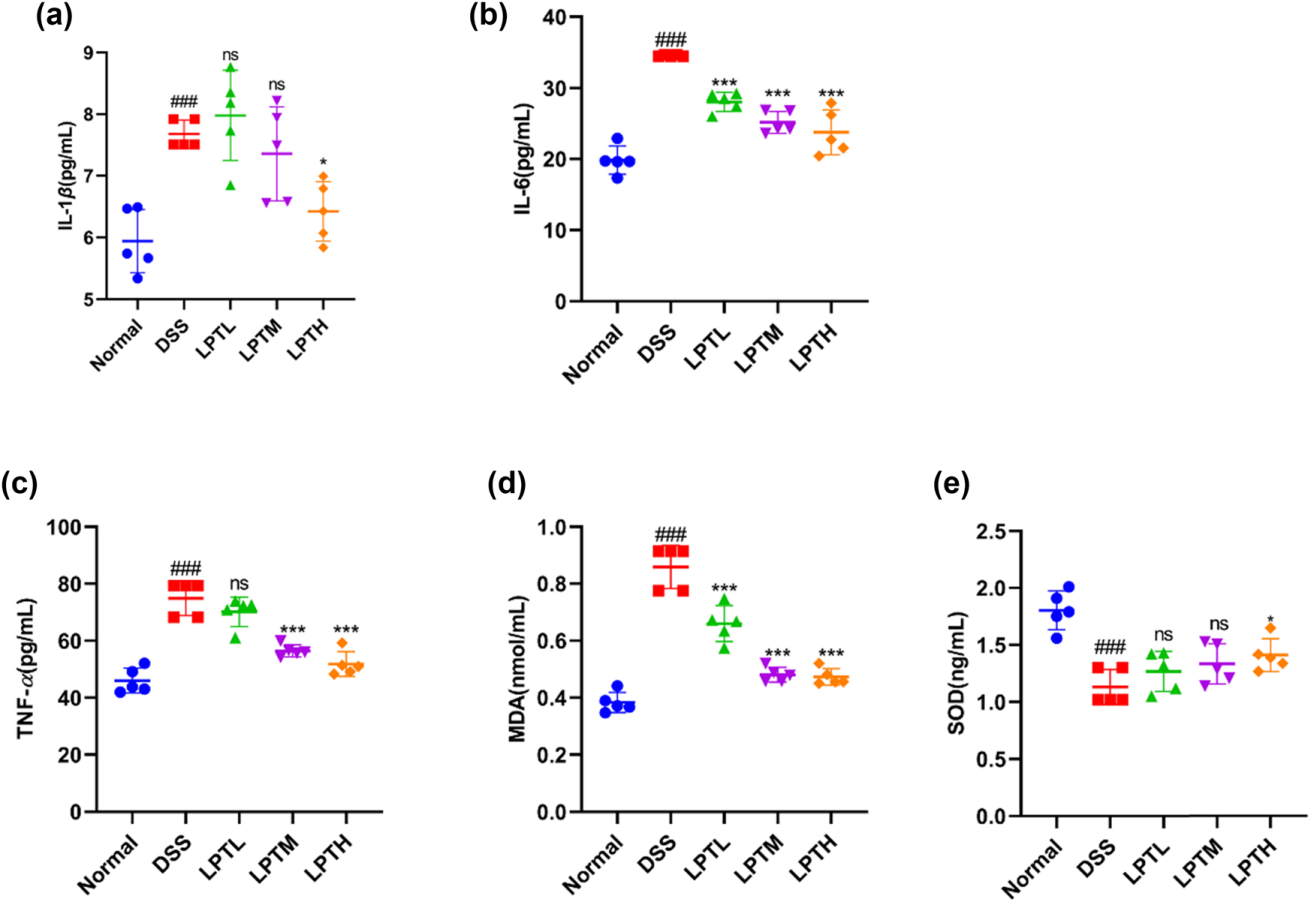
The ameliorative effect of LPTAE on inflammatory and oxidative stress responses in rats with DSS-induced colitis. (a) Concentration of IL-1*β* in serum, (b) concentration of IL-6 in serum, (c) concentration of TNF-*α* in serum, (d) concentration of MDA in serum, and (e) concentration of SOD in serum. All data are presented as mean ± standard deviation (*n* = 5). ###*p* < 0.001 vs the Normal group; *p < 0.05, ***p < 0.001 vs the DSS group; ns denotes no statistically significant difference when compared with the DSS group.

We assessed the systemic immune-inflammatory response in rats by measuring the serum levels of IL-1*β*, IL-6, and TNF-*α*, and evaluated oxidative stress by detecting the levels of MDA and SOD (Figure 2a–e). Compared to the Normal group, the intake of DSS significantly increased the serum levels of IL-1*β*, IL-6, TNF-*α*, and MDA, while decreasing SOD levels (*p* < 0.001). However, intervention with LPTAE resulted in a dose-dependent reduction of IL-1*β*, IL-6, TNF-*α*, and MDA levels in the serum and an increase in SOD levels, with the highest dose of LPTAE showing the most pronounced effects (*p* < 0.05, *p* < 0.001). These findings suggest that the anti-inflammatory and antioxidant properties of LPTAE may contribute to its ability to alleviate symptoms of DSS-induced acute experimental UC.

### 3.3 Effect of LPTAE intervention on the gut microbiota of rats with DSS-induced UC

#### 3.3.1 Analysis of gut microbiota diversity

**Figure 3:**
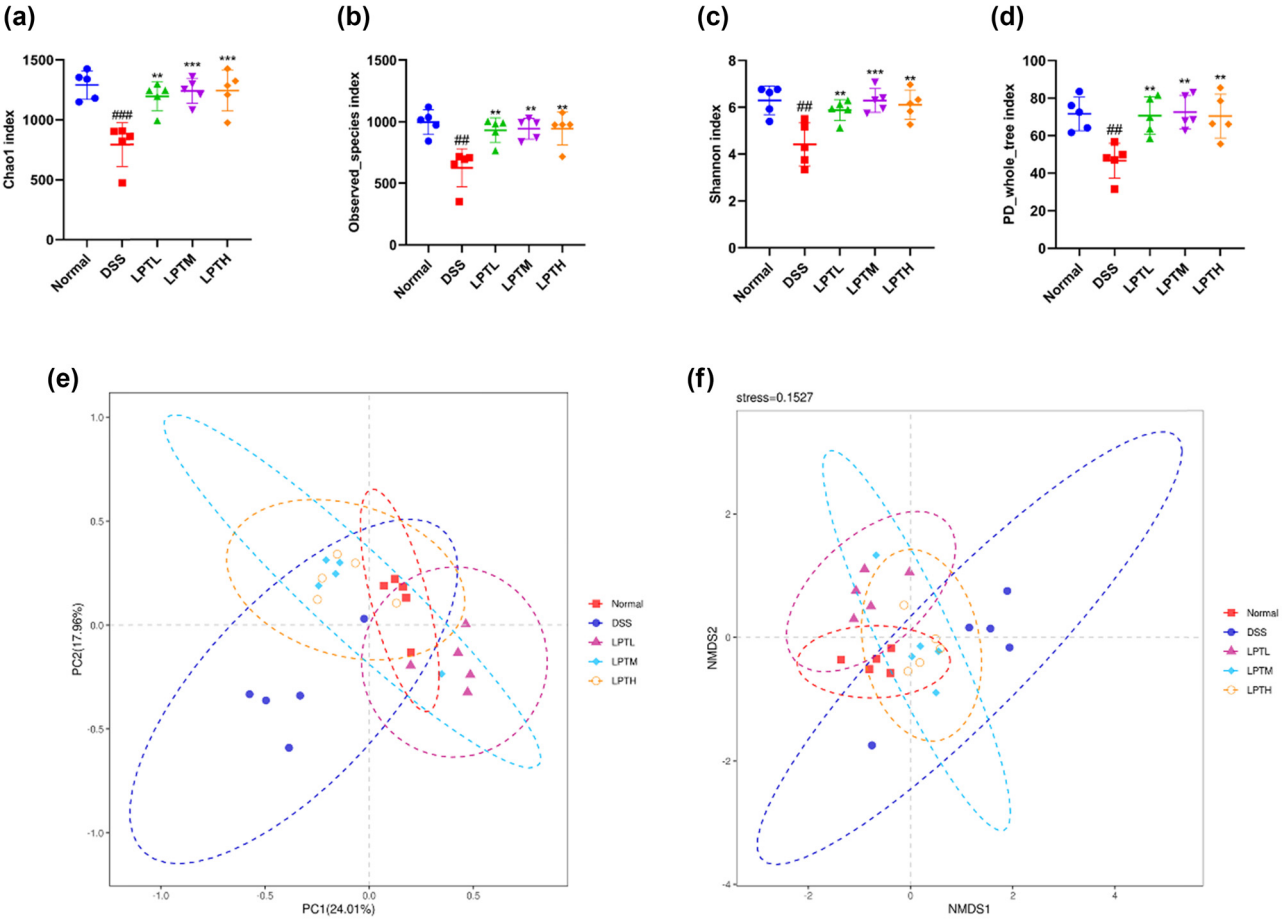
The effect of LPTAE intervention on the gut microbial community diversity of rats with DSS-induced colitis. (a–d) Comprehensive analysis of *α* diversity. (e) PCA at the OTU Level, with each symbol depicting an individual rat. (f) NMDS analysis at the OTU Level, where each symbol signifies an individual rat. ##*p* < 0.01, ###*p* < 0.001 vs the Normal group; **p < 0.01, ***p < 0.001 vs the DSS group.

We evaluated the richness and diversity of the gut microbiota using *α*-diversity indices. The results from 16S rDNA gene sequencing indicated that, compared to the Normal group, the DSS group exhibited a significant decrease in the Chao 1 index, Observed Species index, Shannon index, and PD_whole_tree index (*p* < 0.01, *p* < 0.001). Conversely, when comparing the DSS group with the LPTL, LPTM, and LPTH groups, we observed significant increases in the Chao 1 index, Observed Species index, Shannon index, and PD_whole_tree index (*p* < 0.01, *p* < 0.001) (Figure 3a–d). These findings suggest that DSS-induced colitis in rats leads to a decrease in the *α*-diversity of the gut microbiota, which can be reversed by LPTAE treatment. We also assessed the similarity and dissimilarity in gut microbiota compositions using *β*-diversity indices. PCA results showed a clear separation between the bacterial profiles of the DSS group and the Normal group, with the DSS group samples being the farthest from the Normal group. In contrast, the bacterial profiles of the LPTL, LPTM, and LPTH groups were closer to those of the Normal group, with the LPTH group being the closest (Figure 3e). NMDS analysis further revealed that the Normal group and DSS group were the most distant in terms of clustering, while the LPTL, LPTM, and LPTH groups clustered more closely with the Normal group (Figure 3f). These results indicate that DSS-induced colitis in rats results in significant differences in the composition and structure of the gut microbiota compared to the Normal group and that LPTAE treatment within the tested dose can improve these differences, shifting the microbial community composition and structure towards normality.

#### 3.3.2 Analysis of gut microbiota composition

**Figure 4:**
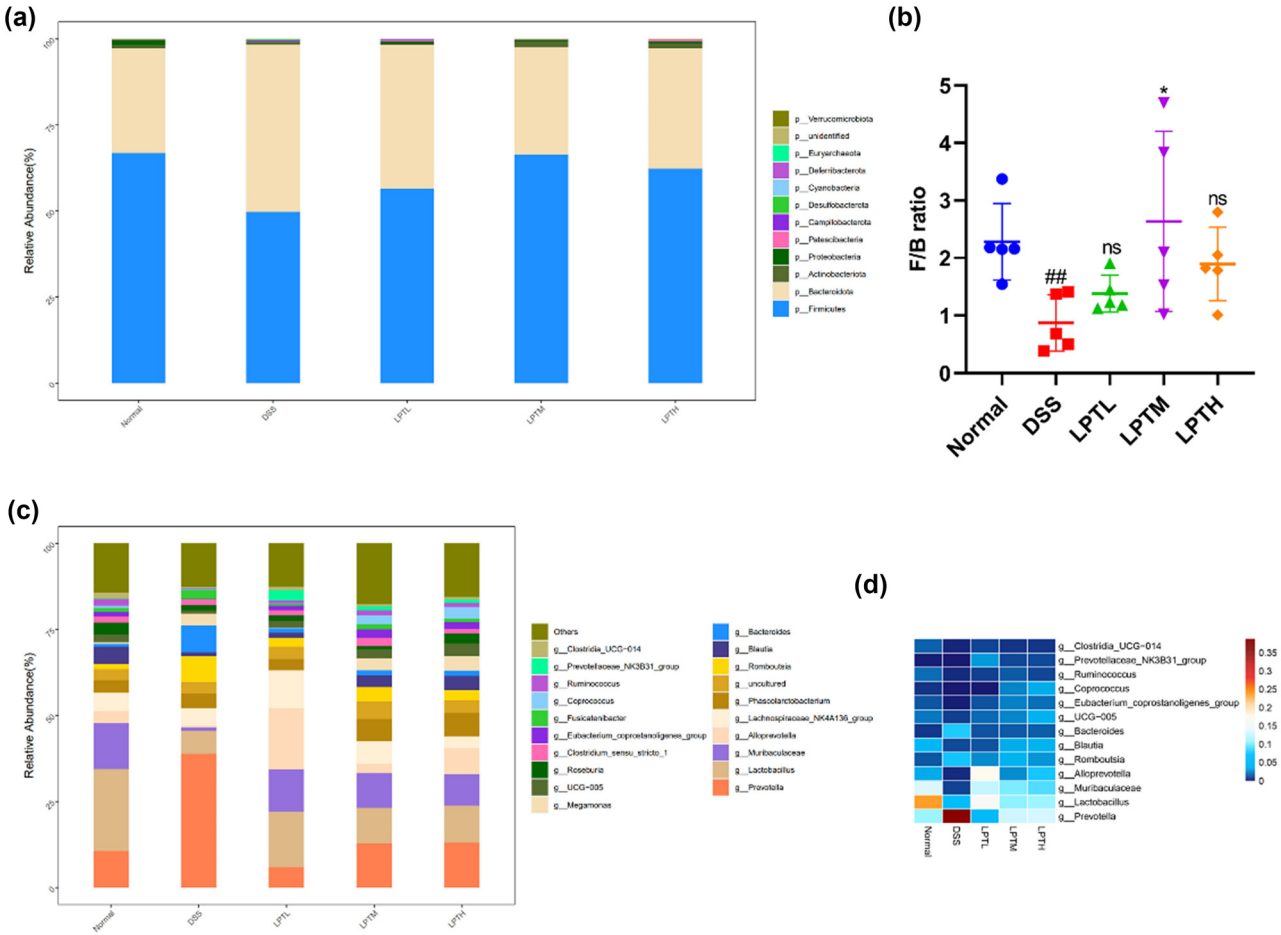
The effect of LPTAE intervention on the composition of gut microbial communities in Rats with DSS-induced colitis. (a) The bar plot of community composition on the Phylum level. (b) The Firmicutes-to-Bacteroidetes ratio. (c) The bar plot of community composition on the genus level. (d) The heatmap depicting the gut microbiota at the genus level. Statistical significance is denoted as follows: ##*p* < 0.01, vs the Normal group; *p < 0.05, vs the DSS group; ns denotes no statistically significant difference when compared with the DSS group.

To further investigate the impact of LPTAE on the gut microbiota community, we analyzed the composition and relative abundance of the gut microbiota at both the phylum and genus levels. The gut microbiota of rats in each group was primarily composed of Firmicutes, Bacteroidota, Actinobacteriota, and Proteobacteria at the phylum level, with Firmicutes and Bacteroidota being the dominant phyla. As shown in Figure 4a, compared to the Normal group, the DSS group exhibited a decrease in the relative abundance of Firmicutes and an increase in the relative abundance of Bacteroidota. In contrast, compared to the DSS group, the LPTL, LPTM, and LPTH groups showed an increase in the relative abundance of Firmicutes and a decrease in the relative abundance of Bacteroidota, with the LPTM group displaying a gut microbiota composition that was closer to that of the Normal group. Next, we calculated and compared the Firmicutes-to-Bacteroidota (F/B) ratio based on the relative abundance values of these two phyla to visualize the changes more clearly (Figure 4b). We found that, compared to the Normal group, the DSS group had a significantly decreased F/B ratio (*p* < 0.01). When compared to the DSS group, the LPTL (*p* > 0.05), LPTM (*p* < 0.05), and LPTH (*p* > 0.05) groups showed an increasing trend in the F/B ratio. At the genus level, we visualized the average relative abundance of the top 20 bacterial genera using a bar chart (Figure 4c) and created a heatmap based on color changes to reflect the relative abundance of 13 bacterial genera with significant differences between groups (Figure 4d). We found that, compared to the Normal group, the DSS group had increased relative abundances of *Prevotella*, *Romboutsia*, and *Bacteroides*, while the relative abundances of *Lactobacillus*, Muribaculaceae, *Alloprevotella*, *Blautia*, UCG-005, *Eubacterium*_*coprostanoligenes*_group, *Coprococcus*, *Ruminococcus*, Prevotellaceae_NK3B31_group, and *Clostridia*_UCG-014 were decreased. Compared with the DSS group, the LPTL, LPTM, and LPTH groups showed a decreasing trend in the relative abundances of *Prevotella*, *Romboutsia*, and *Bacteroides*, with the LPTL group having relative abundances of these genera closer to those of the Normal group. At the same time, the relative abundances of *Lactobacillus*, Muribaculaceae, *Alloprevotella*, *Blautia*, UCG-005, *Eubacterium*_*coprostanoligenes*_group, *Coprococcus*, *Ruminococcus*, Prevotellaceae_NK3B31_group, and *Clostridia*_UCG-014 increased in the LPTL, LPTM, and LPTH groups, approaching those of the Normal group. The relative abundances of *Lactobacillus*, Muribaculaceae, *Alloprevotella*, Prevotellaceae_NK3B31_group, and *Clostridia*_UCG-014 increased most significantly in the LPTL group, while *Eubacterium*_*coprostanoligenes*_group and *Ruminococcus* saw the greatest increase in the LPTM group. Meanwhile, *Blautia*, UCG-005, and *Coprococcus* displayed the most significant increases in the LPTH group. These results indicate that at both the phylum and genus levels, the three tested doses of LPTAE can reverse the gut microbiota imbalance induced by DSS, making the composition of the gut microbiota more similar to that of the Normal group.

#### 3.3.3 Analysis of differential gut microbiota

**Figure 5:**
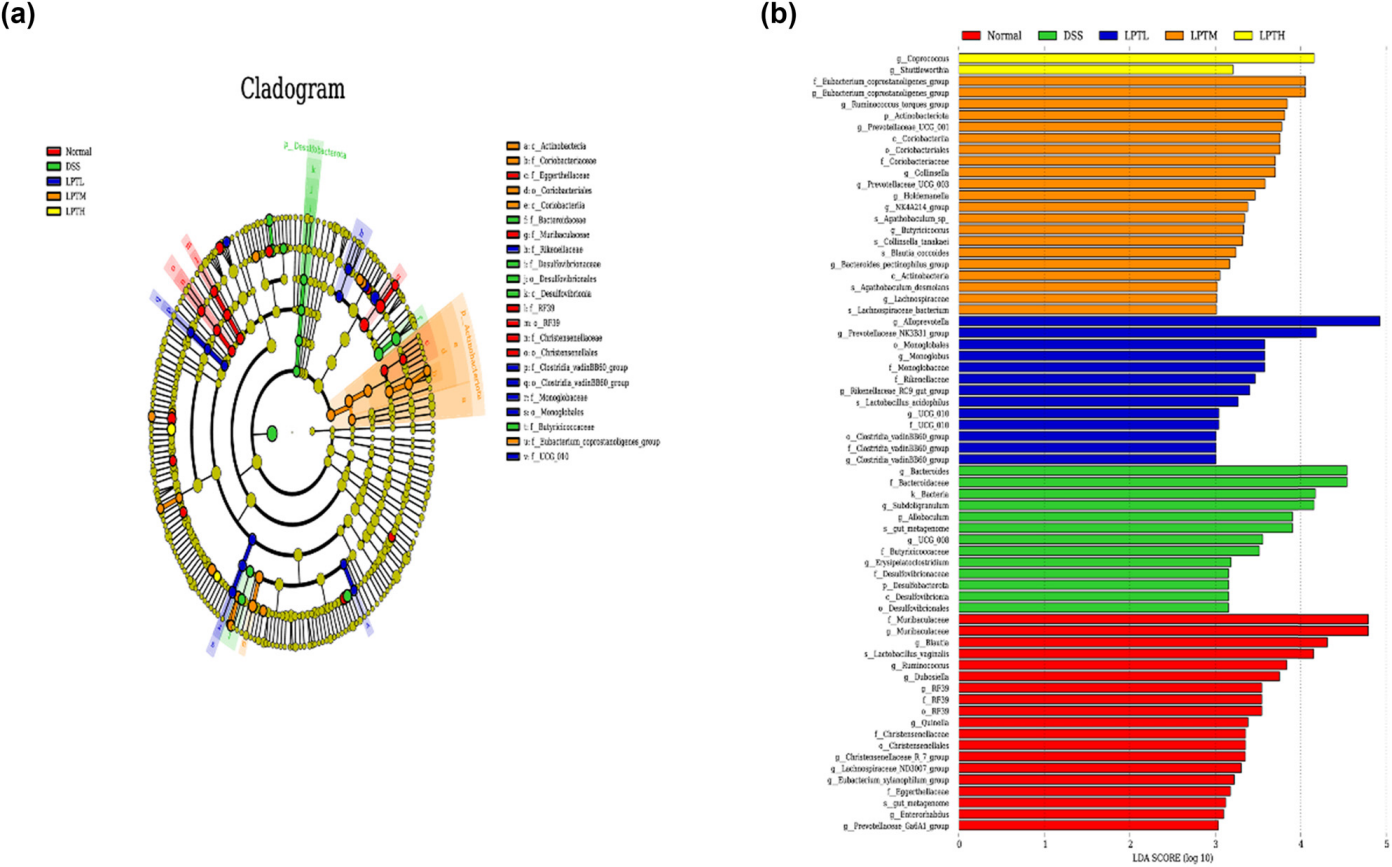
Identification of microbial communities with significant differences using LEfSe analysis. (a) Taxonomic cladogram acquired through LEfSe analysis. (b) Distribution histogram constructed on the basis of LDA, employing a threshold of 4.0 for the log LDA score. This figure portrays species exhibiting notably distinct abundances across various groups, with the length of the bars in the histogram indicative of the magnitude of the impact associated with the significantly differing species.

Using LEfSe analysis, we identified microbial communities with significant differences among the five groups, which serve as important microbial markers. As illustrated in Figure 5a and b, when the LDA > 4 and *p* < 0.05, the dominant microbiota in the intestines of rats in the Normal group included f_Muribaculaceae, g_Muribaculaceae, g_*Blautia*, and s_*Lactobacillus vaginalis*. In the DSS group, the dominant microbiota was identified as k_Bacteria, f_Bacteroidaceae, g_*Bacteroides*, and g_*Subduligranulum* genus. In the LPTL group, the predominant microbiota were g_*Alloprevotella* and g_Prevotellaceae_NK3B31_group. For the LPTM group, the dominant microbiota included f_*Eubacterium*_*coprostanoligenes*_group and g_*Eubacterium*_*coprostanoligenes*_group. Finally, in the LPTH group, the primary microbiota was identified as g_*Coprococcus*. These findings suggest that LPTAE can alter the composition and structure of gut microbiota in rats with DSS-induced UC.

### 3.4 Effect of LPTAE intervention on the content of SCFAs in the colonic contents of DSS-induced colitis rats

**Figure 6:**
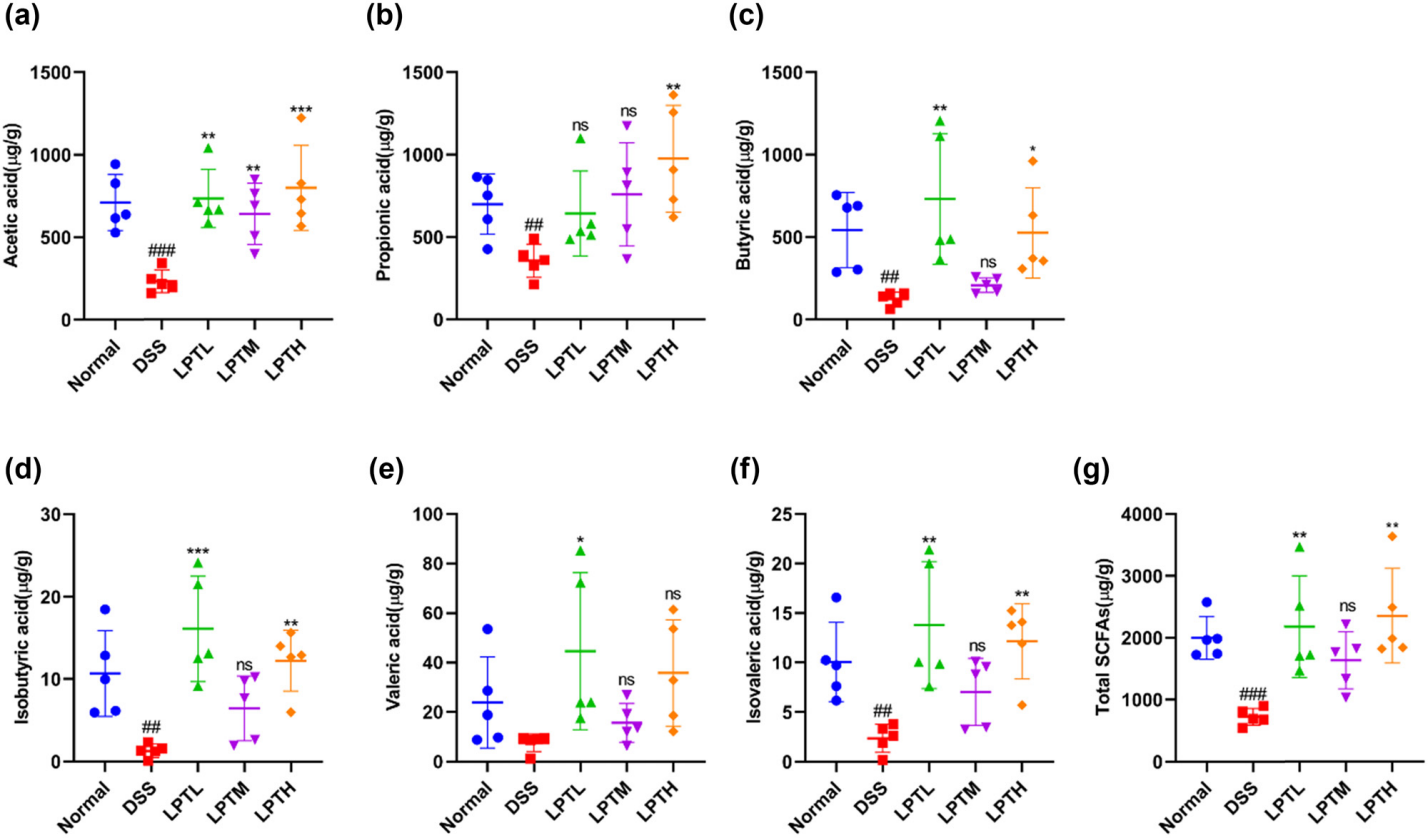
The effect of LPTAE intervention on enhancing the levels of SCFAs in the colonic contents of rats with DSS-induced colitis. (a) Concentration of acetic acid. (b) Concentration of propionic acid. (c) Concentration of butyric acid. (d) Concentration of isobutyric acid. (e) Concentration of valeric acid. (f) Concentration of isovaleric acid. (g) Total concentration of SCFAs. The results are expressed as the mean ± standard deviation (*n* = 5). Statistical significance is denoted as follows: ##*p* < 0.01, ###*p* < 0.001, vs the Normal group; *p < 0.05, **p < 0.01, ***p < 0.001, vs the DSS group; ns denotes no statistically significant difference when compared with the DSS group.

We analyzed the content of SCFAs in the colonic contents using targeted metabolomics. As shown in Figure 6, the DSS group exhibited significantly decreased levels of acetic acid, propionic acid, butyric acid, isobutyric acid, isovaleric acid, and total SCFAs when compared to the Normal group (*p* < 0.01, *p* < 0.001). While the level of valeric acid also decreased, this change was not statistically significant (*p* > 0.05). In comparison to the DSS group, the LPTL group showed significantly increased levels of acetic acid, butyric acid, isobutyric acid, valeric acid, isovaleric acid, and total SCFAs (*p* < 0.05, *p* < 0.01, *p* < 0.001). The level of propionic acid increased as well, but this difference was not statistically significant (*p* > 0.05). In the LPTM group, acetic acid levels showed a significant increase compared to the DSS group (*p* < 0.01). However, the increases in propionic acid, butyric acid, isobutyric acid, valeric acid, isovaleric acid, and total SCFAs were not statistically significant (*p* > 0.05). In the LPTH group, acetic acid, propionic acid, butyric acid, isobutyric acid, isovaleric acid, and total SCFAs had significantly increased levels compared to the DSS group (*p* < 0.05, *p* < 0.01, *p* < 0.001). Although the level of valeric acid also increased, this change was not statistically significant (*p* > 0.05). These results indicate that DSS intake can reduce the production of SCFAs, which are metabolites of the gut microbiota in rats. However, LPTAE intervention can reverse the decrease in SCFAs in the intestines of colitis rats. Increasing SCFA levels in the intestine may serve as the basis for its anti-inflammatory and antioxidant effects.

### 3.5 Spearman correlation analysis between gut microbiota, SCFAs, and colitis-related parameters

**Figure 7:**
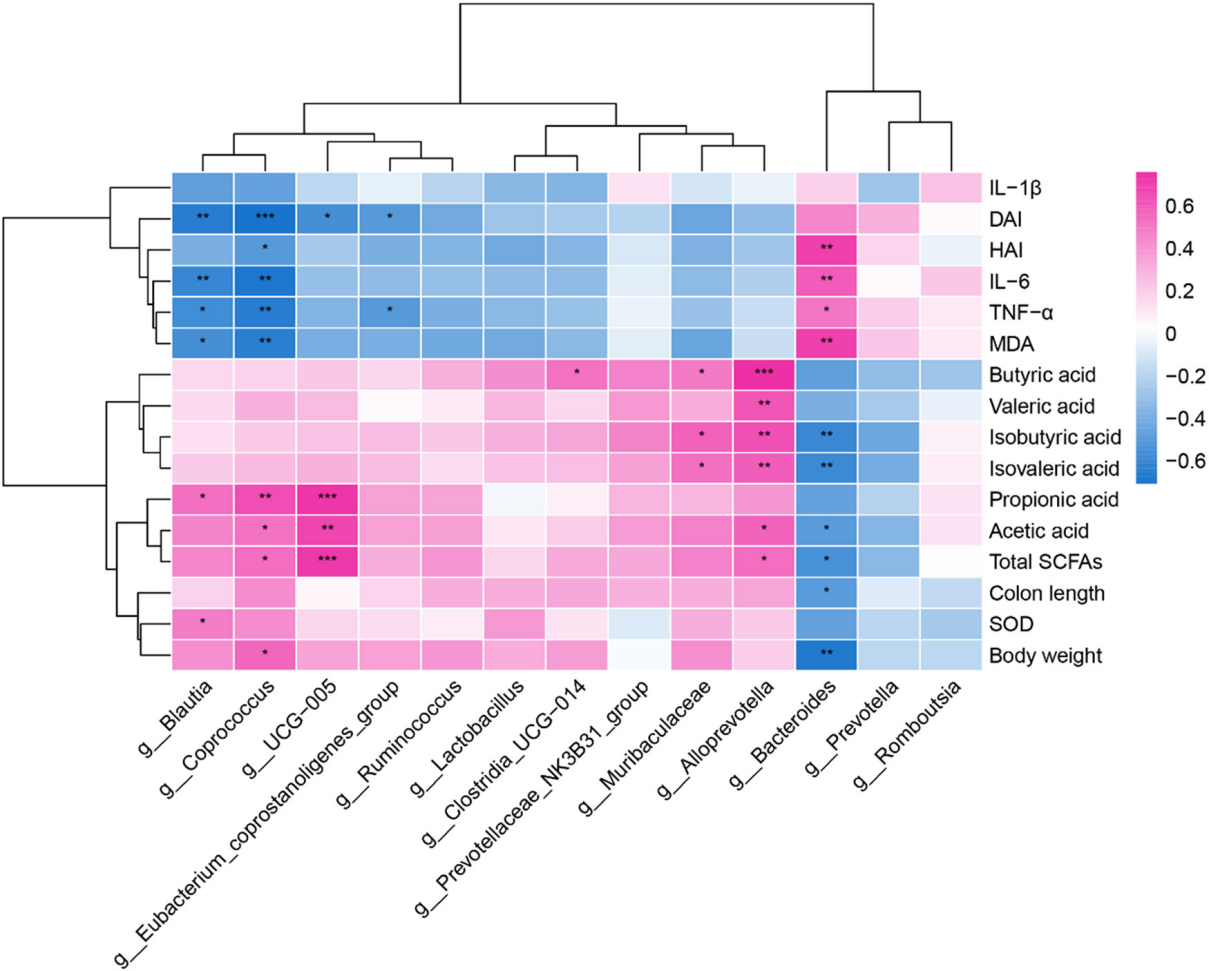
The heatmap of Spearman correlation coefficients between gut microbiota, SCFAs, and colitis-related parameters. Red is positive correlation, blue is negative correlation, *p < 0.05, **p < 0.001, ***p < 0.0001.

A Spearman correlation analysis was performed to examine the relationships among the 13 significantly different gut microbiota at the genus level, SCFAs, and colitis-related parameters. This analysis explored the connections between gut microbiota, SCFAs, IL-1*β*, IL-6, TNF-*α*, MDA, SOD, DAI, HAI, colon length, and body weight. The results are illustrated in Figure 7, where red indicates a positive correlation and blue indicates a negative correlation. In summary, the dominant genera in the DSS group were positively correlated with worsening colitis, while those in the LPTL, LPTM, and LPTH groups were positively correlated with the alleviation of colitis. Specifically: *Bacteroides* showed a significant negative correlation with total SCFAs, acetic acid, isobutyric acid, isovaleric acid, colon length, and body weight (*p* < 0.05, *p* < 0.01). It also exhibited a significant positive correlation with IL-6, TNF-*α*, MDA, and HAI (*p* < 0.05, *p* < 0.01). *Blautia* was significantly positively correlated with propionic acid and SOD (*p* < 0.05) while showing a significant negative correlation with IL-6, TNF-*α*, MDA, and DAI (*p* < 0.05, *p* < 0.01). *Coprococcus* demonstrated a significant positive correlation with total SCFAs, acetic acid, propionic acid, and body weight (*p* < 0.05, *p* < 0.01), and a significant negative correlation with IL-6, TNF-*α*, MDA, DAI, and HAI (*p* < 0.05, *p* < 0.01). UCG-005 showed a significant positive correlation with total SCFAs, acetic acid, and propionic acid (*p* < 0.01, *p* < 0.001), along with a significant negative correlation with DAI (*p* < 0.05). *Eubacterium*_*coprostanoligenes*_group was significantly negatively correlated with TNF-*α* and DAI (*p* < 0.05). *Clostridia*_UCG-014 demonstrated a significant positive correlation with butyric acid (*p* < 0.05). Muribaculaceae was significantly positively correlated with butyric acid, isobutyric acid, and isovaleric acid (*p* < 0.05). *Alloprevotella* showed a significant positive correlation with total SCFAs, acetic acid, butyric acid, isobutyric acid, valeric acid, and isovaleric acid (*p* < 0.05, *p* < 0.01, *p* < 0.001). These findings suggest that LPTAE may alleviate colitis by regulating the composition of gut microbiota, promoting the growth of beneficial bacteria, and inhibiting the growth of harmful bacteria.

## 4 Discussion

In China, traditional Chinese medicine, which is rich in active ingredients such as plant polysaccharides, polyphenols, and alkaloids, has played an important role in the treatment of UC [18]. Active compounds extracted from natural plants, like *Pulsatilla chinensis* saponins, Rattan pepper polysaccharide, and green tea polyphenols, have been reported to effectively alleviate DSS-induced colitis in mice [19,20,21]. They do this by regulating the gut microbiota and reducing oxidative stress and inflammatory responses. Zhou et al. [22] investigated the therapeutic effect of Pu’er tea extract on DSS-induced UC in mice and found that both 100 and 300 mg/kg doses of the extract improved DSS-induced abnormalities in gut microbiota. The treatment significantly alleviated symptoms such as weight loss, bloody diarrhea, loose stools, colonic atrophy, splenomegaly, and colonic pathological damage. Additionally, they observed a downregulation of pro-inflammatory cytokines (IL-1*β*, IL-6, and TNF-*α*) and an increase in the expression of the tight junction protein ZO-1. Similarly, Zhang et al. [23] discovered that Tieguanyin tea extract could correct the gut microecological imbalance caused by DSS. The extract downregulated pro-inflammatory cytokines (IL-4, IFN-*γ*, IL-17, and TGF-*β*) and upregulated the level of the anti-inflammatory cytokine IL-10, significantly reducing the severity of colitis and improving colonic morphology. In our study, we also found that LPTAE, when administered within the tested dose (100, 150, 250 mg/kg), alleviated DSS-induced symptoms such as bloody diarrhea, loose stools, weight loss, colonic swelling, and shortening in rats with colitis. It reduced the DAI, decreased the infiltration of inflammatory cells, and lowered HAI scores in colonic tissue. Furthermore, it promoted tissue repair and mitigated colonic inflammation.

Inflammatory responses and oxidative stress are pathophysiological processes associated with UC. These conditions manifest as abnormal activation of the immune system, an imbalance in the Th17/Treg axis, heightened activation of macrophages and monocytes, and a significant influx of neutrophils and lymphocytes, abnormal activation of various signaling pathways, such as NF-κB, JAK–STAT, MAPK–ERK, and PI3K–Akt, triggers an inflammatory cascade that results in markedly increased levels of pro-inflammatory cytokines, including IL-1*β*, IL-6, and TNF-*α* in the serum [24,25,26]. By measuring the levels of IL-1*β*, IL-6, and TNF-*α*, one can comprehensively assess the severity of inflammation in the body [25,27]. Additionally, MDA, a product of lipid peroxidation in cell membranes, serves as an indicator of oxidative stress and lipid peroxidation [28]. SOD, the primary free radical scavenger in the body, provides an indirect measure of the body’s capacity to scavenge oxygen radicals, based on its activity [29]. A combined assessment of MDA and SOD levels offers a thorough evaluation of oxidative damage and antioxidant capacity within the body. Findings from this study indicate that DSS-induced colitis in rats leads to significantly increased levels of IL-1*β*, IL-6, TNF-*α*, and MDA, along with a notable decrease in SOD levels, suggesting severe oxidative damage and inflammatory responses. Treatment with LPTAE reduces the levels of IL-1*β*, IL-6, TNF-*α*, and MDA, increases SOD levels, and mitigates inflammatory responses and oxidative stress damage. The tea polyphenols, theanine, and tea pigments present in LPTAE may be the primary contributors to these effects [5,9,11,30,31,32]. They are thought to exert antioxidant and anti-inflammatory actions through NF-κB, JAK–STAT, MAPK–ERK, and PI3K–Akt signaling pathways, ultimately downregulating IL-1*β*, IL-6, and TNF-*α* levels and alleviating inflammation [33,34,35].

The normal structure and composition of the gut microbiota are essential for maintaining intestinal homeostasis, which is crucial for overall body health. Imbalances in intestinal microecology are closely linked to the occurrence and progression of diseases, such as UC [36]. Dysregulation of the intestinal microecology can disrupt immune homeostasis and the epithelial barrier function of the intestine. This disruption occurs through microbial metabolites that increase the permeability of epithelial cells, allowing harmful microorganisms to invade and colonize more easily, which in turn heightens the susceptibility to colitis [22]. Patients with UC often exhibit reduced microbial diversity in their intestines [26]. Our analysis of the colonic microbiota, utilizing 16S rDNA gene sequencing technology, supports this observation. Our research indicates that LPTAE intervention can positively impact both the *α*-diversity and *β*-diversity of the gut microbiota in rats with colitis. This intervention helps reverse the reduction in gut microbiota diversity in these rats, corrects the dysbiosis of the gut microbiota, and brings the composition and structure of the microbial community in colitis rats closer to that of the normal control group. These findings are consistent with the results reported by Zhou et al. [22].

Previous studies have indicated that Firmicutes and Bacteroidota make up over 90% of the relative abundance in the gut microbiota of both humans and animals [37]. Changes in the F/B ratio can reflect the balance of the intestinal microecology, with a decrease in this ratio being associated with IBD [38]. In our study, we observed that Firmicutes and Bacteroidota dominated the gut microbiota of rats across all groups, but there were variations in their relative abundance. Notably, rats with DSS-induced colitis exhibited a significant decrease in the F/B ratio. However, after intervention with LPTAE at tested doses (100, 150, 250 mg/kg), the declining trend in the F/B ratio was reversed, approaching the levels observed in the normal control group. The intestinal microecological imbalance in UC is characterized by the reduction or growth inhibition of beneficial and commensal bacteria, alongside an increase in harmful bacteria [36]. At the genus level, we found that the relative abundance of *Bacteroides*, *Prevotella*, and *Romboutsia* significantly increased in the intestines of colitis rats, while the abundance of ten bacterial groups, including *Lactobacillus*, Muribaculaceae, *Alloprevotella*, *Blautia*, UCG-005, *Eubacterium*_*coprostanoligenes*_group, *Coprococcus*, *Ruminococcus*, the Prevotellaceae_NK3B31_group, and *Clostridia*_UCG-014, significantly decreased. Previous research has shown that the abundance of *Bacteroides*, *Prevotella*, and *Romboutsia* significantly rises in DSS-induced UC. These bacteria are considered opportunistic pathogens and are linked to the onset of colitis [12]. An increase in *Bacteroides* and *Prevotella*, both belonging to the Bacteroidota phylum, is typically thought to exacerbate inflammatory responses [39]. Additionally, it has been observed that the population of SCFA-producing bacteria is reduced in UC [40]. *Lactobacillus*, Muribaculaceae, *Alloprevotella*, and Prevotellaceae_NK3B31_group are common probiotics vital for maintaining normal intestinal pH, the barrier function of the intestinal mucosa, and resistance to inflammation [41]. *Lactobacillus* plays a regulatory role in abnormal immune responses during intestinal inflammation, showing positive correlations with tight junction proteins and negative correlations with pro-inflammatory cytokines [42]. *Alloprevotella* primarily produces butyrate, which helps mediate the host’s anti-inflammatory and intestinal barrier-protective effects [43]. *Blautia* is recognized as an intestinal probiotic. Studies indicate that *Blautia* can reduce levels of inflammatory cytokines such as IL-1*β*, IL-6, and TNF-*α* by regulating the TLR4/NF-κB signaling pathway, thereby inhibiting inflammatory responses, alleviating oxidative stress, and protecting the intestinal barrier [44]. Zhao et al. found that theabrownin, derived from Pu’er tea, can improve DSS-induced colitis by increasing the abundance of bacteria such as *Eubacterium*_*coprostanoligenes*_group and inhibiting the TLR2 and TLR4 signaling pathways, suggesting its probiotic role in the intestine [45]. *Ruminococcus* and a specific genus (noted as UCG-005) belong to the Ruminococcaceae family and are primarily involved in degrading indigestible dietary fiber and producing butyrate, which provides energy to colonic epithelial cells, preserves intestinal mucosal integrity, regulates the immune system, and combats inflammation [46]. *Coprococcus*, mainly found in feces, is another significant producer of butyrate and acts as a microbial marker for assessing intestinal health. It exerts anti-inflammatory effects by lowering concentrations of pro-inflammatory cytokines (TNF-*α*, IL-1*β*, and IL-6) and increasing levels of anti-inflammatory cytokines (IL-4, IL-5, and IL-10), while also restoring the expression of tight junction proteins like claudin-1, occludin, and ZO-1 [47]. *Clostridia*_UCG-014, a commensal bacterium, is thought to promote the growth of beneficial bacteria, inhibit harmful bacteria, and facilitate the digestion and absorption of nutrients by regulating intestinal microecological balance [19]. In this study, we observed a decreasing trend in the relative abundance of harmful bacteria, including *Bacteroides*, *Prevotella*, and *Romboutsia*, following treatment with low, medium, and high doses of LPTAE. In contrast, the relative abundance of beneficial and commensal bacteria such as *Lactobacillus*, Muribaculaceae, *Alloprevotella*, *Blautia*, *Eubacterium coprostanoligenes* group, *Coprococcus*, *Ruminococcus*, the Prevotellaceae NK3B31 group, and *Clostridia* UCG-014 showed varying degrees of increase. LEfSe analysis, a method used to identify dominant species among different groups, indicated that *Alloprevotella* and the Prevotellaceae NK3B31 group, which produce SCFAs, along with *Eubacterium coprostanoligenes* group and *Coprococcus*, were the dominant bacterial groups in the low, medium, and high dose treatment groups, respectively. This further confirms that LPTAE can promote the growth of beneficial bacteria, inhibit the growth of harmful bacteria, and play a significant role in regulating the balance of intestinal microecology.

SCFAs in the intestine are the main metabolites produced by the gut microbiota. They serve as important energy sources and functional regulators for intestinal epithelial cells. SCFAs primarily include acetic acid, propionic acid, butyric acid, isobutyric acid, valeric acid, and isovaleric acid. Among these, acetic acid, propionic acid, and butyric acid account for over 95% of the total SCFAs [48], particularly butyric acid, which exhibits significant anti-inflammatory and antioxidant effects. Research has shown that the levels of SCFAs in the intestinal contents of patients with UC are significantly reduced, indicating a close relationship between SCFAs and the occurrence and progression of UC [18]. Consequently, SCFAs can be seen as a bridge between the gut microbiota and UC. In this study, we observed that the levels of SCFAs in the intestines of rats with colitis decreased significantly, except for valeric acid, which showed a less pronounced decrease. The other SCFAs, including acetic acid, propionic acid, and butyric acid, displayed notable reductions. SCFAs can bind to G protein-coupled receptors GPR41, GPR43, GPR109, and others on the surfaces of intestinal epithelial cells and immune cells, they regulate intestinal epithelial barrier function, immune responses, production of reactive oxygen species in the intestine, inflammatory responses, and colonic motility through various signaling pathways, including NF-κB, TLR/NF-κB/HDAC, and AhR/IL-22 [40]. Importantly, there is a bidirectional communication between SCFAs and the gut microbiota. SCFAs are produced through the fermentation of dietary fiber by the gut microbiota, and in turn, they function as regulators of the microbiome, helping to maintain the balance of intestinal microecology and homeostasis of the internal environment [49]. Our research found that after intervention with the tested doses (100, 150, and 250 mg/kg) of LPTAE, the decreasing trend of SCFAs in the intestines of rats with colitis was reversed to varying degrees. This indicates that LPTAE may help correct the metabolic imbalance of intestinal SCFAs, which could be the basis for its anti-inflammatory and antioxidant effects.

The gut microbiota often regulates the host’s biological functions through its metabolites. In our study, we found that the dominant bacterial genera in the DSS group were negatively correlated with SCFAs, colon length, and body weight, while they showed positive correlations with inflammatory cytokines, MDA, and HAI. In contrast, the dominant bacterial genera following the LPTAE intervention were positively correlated with SCFAs, colon length, and body weight, while negatively correlated with inflammatory cytokines, MDA, and HAI. As noted earlier, the dominant bacterial genera in the DSS group are primarily associated with the onset and progression of colitis. On the other hand, the dominant genera after LPTAE intervention are more likely to be probiotics and commensal bacteria that produce SCFAs. These findings suggest that the gut microbiota imbalance in rats with colitis can be linked to a metabolic imbalance of SCFAs. LPTAE appears to rectify this metabolic imbalance by promoting the growth of SCFA-producing and commensal bacteria in the intestine while inhibiting the colonization of harmful bacteria. Consequently, this intervention exerts anti-inflammatory and antioxidant effects, alleviating the symptoms of colitis.

Our study does have certain limitations. We observed that LPTAE intervention alleviated symptoms of colitis in rats and helped to restore the intestinal microecological balance. However, it remains to be validated whether this effect is mediated by the gut microbiota in the context of UC. Additionally, the molecular mechanisms and signaling pathways through which LPTAE alleviates UC require further investigation. It is important to note that LPTAE is a complex mixture that contains multiple components, such as tea polyphenols, tea polysaccharides, theanine, and alkaloids. At this point, we cannot identify which specific component is responsible for the therapeutic effect on UC. This will necessitate precise isolation and purification of LPTAE, along with further experiments. Furthermore, we need to study whether these mixed components have beneficial or detrimental effects on other organs.

## 5 Conclusions

In summary, our study demonstrates that LPTAE is an effective gut microbiota modulator. By utilizing LPTAE, we can reshape the gut microbiota in UC. This process promotes the growth of probiotics and beneficial bacteria while inhibiting harmful bacteria, thereby maintaining SCFA levels in the intestine. As a result, we observed a reduction in serum pro-inflammatory cytokines (IL-1*β*, IL-6, TNF-*α*) and an improvement in serum oxidative stress markers (MDA, SOD). These changes contribute to alleviating symptoms of UC in rats. Our findings provide essential data for the clinical application and the development of functional foods of Liupao tea.
